# Artificial Intelligence in Medical Education: a Scoping Review of the Evidence for Efficacy and Future Directions

**DOI:** 10.1007/s40670-025-02373-0

**Published:** 2025-04-02

**Authors:** Kody Shaw, Marcus A. Henning, Craig S. Webster

**Affiliations:** Centre for Medical and Health Sciences Education, School of Medicine, University of Auckland, Auckland, New Zealand

**Keywords:** Artificial intelligence, Machine learning, Medical education, Surgical simulation, SDG 4: Quality education

## Abstract

Artificial intelligence (AI) has demonstrated clinical potential, yet its influence on medical education remains limited. This review explores AI applications in medical education, evaluates available evidence and considers future applications. We conducted a scoping review (PubMed, MEDLINE, SCOPUS, Google Scholar; 2010–2022) identifying 42 relevant peer-reviewed articles. Four key themes emerged: surgical skills assessment, radiology training, interactive learning, and text interpretation. Current applications enhance surgical simulation and facilitate interactive learning. These tools may evolve towards comprehensive and individualised educational aids. Despite promising early applications, evidence on educational and clinical outcomes remains limited. Future research should prioritise validated outcomes in larger trials to confirm generalisability and address AI limitations.

## Introduction

The term artificial intelligence (AI) is increasingly used as a synonym for automation or technological advancement, contributing to a lack of unanimity in definition. For this paper, we define AI as computer systems that perform tasks normally requiring human intelligence [1]. Underpinned by complex algorithms and machine learning, AI can analyse vast quantities of data and identify meaningful patterns to inform decisions [2]. AI replicates the quintessentially human cognitive function of integrating information to inform problem solving or decision making [2].

It seems likely that AI will transform many industries in the foreseeable future, with healthcare and education being no exception. This impact is currently being debated but is exemplified by the success of ChatGPT (Open AI, San Francisco, CA, USA) which has become the world’s fastest growing consumer application [3]. This development also highlights the potential applications of AI in medical education, given that ChatGPT has repeatedly demonstrated the capacity to pass the United States Medical Licensing Examination [4, 5]. More significantly, it reliably generated comprehensive clinical reasoning and novel insights in 90% of responses, suggesting a role for AI in medical education [5].

There has been broad application of AI in clinical medicine from radiological diagnosis [6], recognition of ophthalmological conditions [7], identifying dermatological malignancy [8, 9] and accurately predicting clinical deterioration [10]. In an Australasian survey, 71% of clinicians predicted AI will improve practice, while 60% perceived this will occur within 5 years [11]. Clinicians foresee advantages including improving patient access to screening tests and reducing the burden of monotonous work. Anticipated disadvantages include the growing influence of technology companies in healthcare and the potential for errors [11]. The potential influence of AI in medical education was not raised in this survey. However, international surveys indicate that almost 50% of medical students are less interested in certain specialties due to the likely effects of AI [12].

AI has many conceivable applications in medical education including personalised learning adapted to students’ knowledge or learning style, and potentially reducing inequities in education by universalising access to information [13, 14]. The definition of AI can be adapted to medical education to denote an intelligent system that aims to support educational tasks traditionally requiring human educators, including real time assessment of performance and provision of individualised feedback [15]. The quantity of publications discussing AI in medicine has grown rapidly in the past decade [16]. Despite this, AI applications in medical education remain minimally explored [13]. This review evaluates the current prevalence of publications exploring the use of AI in medical education and further aims to identify specific areas for future research.

## Methods

Our scoping review aims to determine how AI has been applied in medical education to date. As a secondary aim we will consider what outcomes have been measured or observed, and what potential future applications exist. We anticipate that data will be heterogenous and thus, unsuitable for quantitative meta-analysis. The scoping review methodology was selected to explore the subject of AI in medical education, provide an overview of available evidence and identify opportunities for future research.

We conducted our review in accordance with the standards outlined in the Preferred Reporting Items for Systematic Reviews and Meta-Analysis (PRISMA) guideline with the extension for Scoping Reviews (PRISMA-ScR) [17]. Four databases (PubMed, MEDLINE, SCOPUS and Google Scholar) were searched from January 2010 to December 2022. The start date of 2010 was selected to focus on the increasing number of publications on this subject over the last decade. The search strategy was formed in conjunction with an experienced research librarian to ensure all relevant records in these databases were identified. The search contained keywords and subject headings such as artificial intelligence, machine learning, and neural networks combined with various medical education terms. The search strategy was originally formulated on the MEDLINE database and then adapted to other databases. An example of the search strategy is shown in Table 1. Additional records were identified by manually searching the reference lists of articles considered for full text review. Duplicate records were removed.

**Table 1:** Search strategy

	Search terms
1	Exp Artificial Intelligence/ OR (artificial adj3 intelligence).mp OR exp Deep Learning/ OR (deep adj3 learn*).mp OR exp Machine Learning/ OR (machine adj3 learn*).mp or exp Neural Networks, Computer/ OR (neural network*).mp OR exp Natural Language Processing/
2	Limit 1 to (english language and full text and humans and yr = “2010 – current”)
3	Exp Education, Medical/ OR (medic* adj3 educat*).mp OR exp Clinical Clerkship/ OR (clinical adj3 educat*).mp OR exp Schools, Medical/ OR (med* adj3 school*).mp OR (med* adj3 stud*).mp OR (med* adj3 train*).mp OR (contin* adj3 med* adj3 educat*).mp OR exp Internship and Residency/ OR exp Education, Medical, Undergraduate/ OR exp Education, Medical, Graduate/ OR exp Teaching Rounds/
4	Limit 3 to (english language and full text and humans and yr = ”2010 – current”)
5	2 and 4

### Inclusion and Exclusion Criteria

Inclusion criteria were established to focus on reports of artificial intelligence in medical education. This review sought to explore AI that enhanced knowledge or skill acquisition in undergraduate or postgraduate medical training. Accordingly, the exclusion criteria removed reports describing clinical applications of AI without an educational objective, and workforce surveys gauging understanding or preparedness for AI, amongst others. See Table 2 for complete inclusion and exclusion criteria. Original articles published in peer-reviewed journals, in English language with full-text availability, were considered for inclusion.

**Table 2:** Eligibility criteria

Inclusion criteria	- Educational intervention using AI as defined in this paper, with relevance to medical education - AI intervention already applied in practice or demonstrated clear empirical future applications - Full text, English language, published between 2010 and 2022
Exclusion criteria	- Did not meet the stated definition of AI in this paper; examples includes automation of processes or virtual reality - Surveys surrounding AI including current understanding, readiness for implementation, perceived future impact of AI - AI automating clinical duties without educational objective - Discussions of medical school curriculum adaptation to AI - Discussion articles theorising future use of AI in medical education - No full text available, outside inclusion dates, not an original article in peer-reviewed journal

The PICO method informed both the search strategy and inclusion criteria [18].

#### Population

Relevant to undergraduate or postgraduate medical education including medicine and dentistry.

#### Intervention

Any AI based educational intervention that meets this papers definition of AI, including machine or deep learning, neural networks or natural language processing.

#### Comparison

Current standard of care for education, non-AI based technological intervention, or no intervention.

#### Outcome

Reported educational outcomes including learner confidence, knowledge or skill acquisition or clinical performance were considered. Given the emerging nature of evidence in this area, a valid future educational application is also considered.

### Data Extraction

**Fig. 1:**
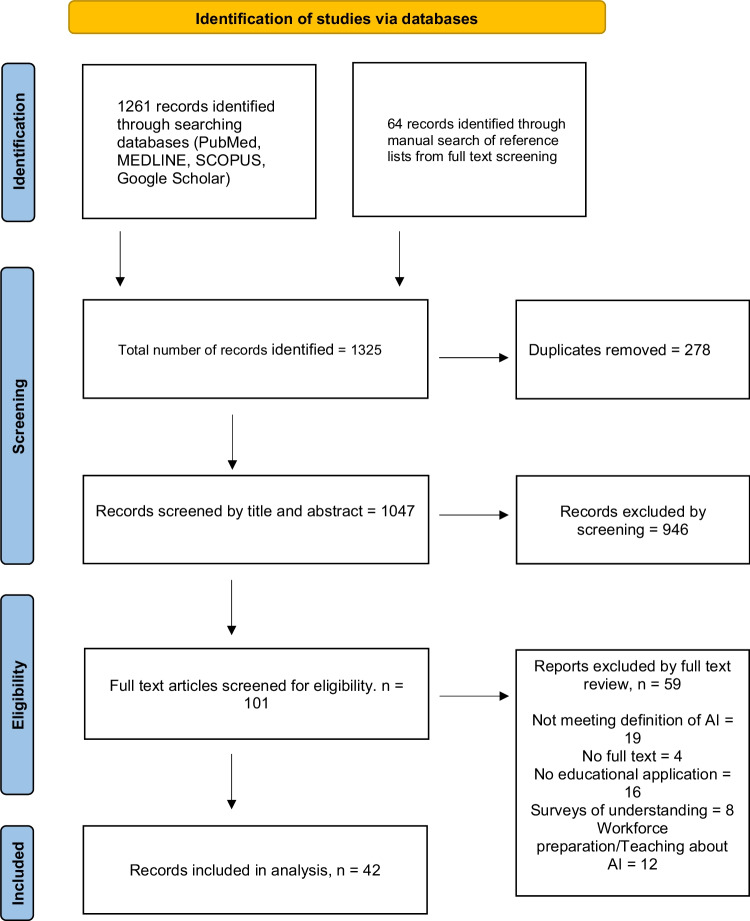
PRISMA flow diagram

Search results were organised using the RefWorks (ProQuest, Michigan, USA) citation management software. The outcome of the search and application of eligibility criteria is demonstrated in Fig. 1. One reviewer (KS) undertook initial screening of all search results by title and abstract to identify those appropriate for full text review. Full records were considered by the first author (KS) for eligibility. All included records and a selection of excluded records were reviewed by the two other authors (MH, CW) to ensure consistency. Data was manually extracted from included reports.

### Data Analysis

All articles were reviewed according to the steps outlined in Fig. 1. After which, the full text of the papers included in the final phase of the scoping review were evaluated to discern whether any meaningful categories could be determined.

## Results

### Search Yield

The search strategy returned 1261 articles, whilst 64 articles were identified through manual searching of reference lists of full texts being reviewed. There were 278 duplicates removed and a further 946 records excluded by screening the title and abstract. Consequently, 101 were subject to full text review and 42 were included. Details of the studies included are depicted in Table 3.

**Table 3:** Included studies

Paper	Publication details	Artificial intelligence application	Outcomes and findings
	**Section 1: Surgical skills**		
1	Alonso-Silverio et al. [19] Mexico	Machine learning assessment of VR augmented box-trainer performance using an artificial neural network	Identifies expert performance with 93.4% accuracy. 90% of participants reported this to be useful
2	Anh et al. [20] Australia	Machine learning algorithm assesses performance using surgical simulation footage from JIGSAWs (JHU-ISI gesture and skill assessment working set) database	The conventional neural network model was 96.9% accurate in differentiating expert from novice
3	Azari et al. [21] USA	Compared automated computer assessment of surgical performance using linear regression model with expert assessment using OSATS (objective structured assessment of technical skill) assessment	Computer assessment correlates highly with expert assessment, with less variability. Computer assessment of intra-operative video is feasible
4	Bissonnette et al. [22] Canada	Machine learning assessment of VR surgical simulation using support vector machines. Provides audiovisual feedback compared to standard	AI differentiates expert from novice with 97.6% accuracy. Identified specific performance metrics for feedback
5	Davids et al. [23] UK	Neural network assessment of video footage of simulation	AI differentiates expert from novice with 84.2% accuracy. Identified specific metrics for feedback
6	Fard et al. [24] USA	Support vector machine assessment of microsurgery simulation	AI differentiates expert from novice with 82.3–89.9% accuracy depending on task. Generates immediate feedback
7	Ismail Fawaz et al. [25] France	Conventional neural network assessment of surgical simulation video from JIGSAWS database	AI differentiates expert from novice with 100% accuracy. Heatmap visual feedback provided
8	Fazlollahi et al. [26] Canada	Machine learning assessment (support vector machine) of VR surgical simulator compared with traditional instruction. Provides real-time video feedback	Trainees acquired skills 2.6 × faster and performed 36% better than those using simulator without AI
9	Fekri et al. [27] Canada	Deep neural network based intelligent tutoring system to teach orthopaedic procedure with haptic guidance	Successful prototype to teach surgical procedure to novices
10	French et al. [28] USA	Machine learning assessment of video from laparoscopic surgical simulator	AI differentiates expert from novice with 85% accuracy 90% of data required to make decision available in first 8 s
11	Funke et al. [29] Germany	Temporal segment neural network assessment of surgical simulation video from JIGSAWS database	AI differentiates expert from novice with 95.1–100% accuracy depending on task
12	Julian and Smith [30] USA	Developed intelligent tutoring system to teach robotic surgery skills. Adaptive difficulty based on performance	Successful development of ITS
13	Khalid et al. [31] Canada	Deep learning assessment of surgical simulation video from JIGSAWS database. Utilised neural network called autoencoder	AI differentiates expert from novice with mean 97% accuracy
14	Kowalewski et al. [32] Germany	Machine learning (neural network, decision forest, decision tree) assessment of laparoscopic simulator performance using kinematic data from wearable armband. Compared with expert assessment using OSATS	AI differentiates expert from novice with 79.5–82.2% accuracy depending on task. AI assessment correlates closely with expert assessment
15	Lavanchy et al. [33] Switzerland	Machine learning assessment of intra-operative video from laparoscopic surgery using convolutional neural network	AI assessment was 87% accurate compared to expert assessment
16	Ledwos et al. [34] Canada	K-nearest neighbour machine learning algorithm assessment of VR surgical simulator to generate learning curves	AI differentiates expert from novice with 90% accuracy. Generated learning curves for trainees to inform and monitor development
17	Mirchi et al. [35] Canada	Machine learning assessment of VR surgical simulator. Provides individualised feedback	AI differentiates expert from novice with 92% accuracy
18	Mirchi et al. [36] Canada	Machine learning and artificial neural network assessment of VR surgical simulator. Provides individualised feedback	AI differentiates expert from novice with 83% accuracy
19	Nakawala et al. [37] Italy	Machine learning intelligent tutoring system to teach thoracocentesis on VR simulator. Compared to expert-led teaching	Non-significant improvement in performance in group that was taught using the ITS vs traditional human-led teaching
20	Nguyen et al. [38] Australia	Deep learning neural network model assessment of surgical simulation video from JIGSAWS database	AI differentiates expert from novice with 98.4–98.7% accuracy
21	Oquendo et al. [39] USA	Machine learning model trained assessment of laparoscopic box trainer simulation using kinematic sensors in tools. Compared to expert assessment using OSATS	AI assessment was 85% correlated with expert assessment
22	Reich et al. [40] Canada	Artificial neural network model assessment of VR surgical simulator	AI differentiates expert from novice with 83% accuracy. Specific feedback to learners based on measured metrics
23	Rhienmora et al. [41] Thailand	AI using Hidden Markov models assessment of VR simulator for dental procedures	AI differentiates expert from novice with 100% accuracy
24	Siyar et al. [42] Iran	Machine learning algorithm applied to VR neurosurgery simulator. Most effective was support vector machine	Distinguishes between expert and novice with accuracy of 86–90%
25	Su Yin et al. [43] Thailand	AI assessment of VR simulator for dental procedures. Compared to VR simulator without AI feedback and traditional teaching	VR simulation with AI feedback group significantly outperformed the other two groups in post training assessment (91.6% vs. 66.3% vs. 72.2%)
26	Uemura et al. [44] Japan	Neural network assessment of laparoscopic simulator using wearable kinematic motion sensors	AI differentiated expert from novice with 79% accuracy
27	Wang and Majewicz Fey [45] USA	Deep convolutional neural network assessment of surgical simulation video from JIGSAWS database	AI differentiates expert from novice with 91.3–95.4% accuracy depending on task. Data needed to make decision available within 1–3 s
28	Watson [46] USA	AI algorithm assessment (support vector machine) of benchtop simulator using wearable kinematic motion sensor	AI differentiates expert from novice with 83% accuracy
29	Winkler-Schwartz et al. [47] Canda	Machine learning assessment of VR surgical simulation. K-nearest neighbour algorithm was the most accurate	AI differentiates expert from novice with 90% accuracy
30	Yang and Shulruf [48] Taiwan	AI intelligent tutoring system teaching suturing to medical students. Compared to standard expert teaching	The AI group demonstrated 25% improved performance vs. 18% in expert taught group. AI group also had improved confidence suturing
	**Section 2: Radiology**		
31	Cheng et al. [49] Taiwan	Deep learning algorithm trained to identify hip fractures on x-ray. Trial of assisted learning with AI algorithm compared to conventional learning	AI assisted learning group significantly improved between tests with 9% higher scores, control group showed no improvement
32	Dao et al. [50] Canada	Machine learning algorithm to interpret chest X-rays. Applied in trial of medical students to assess whether using AI platform for one test improved performance without assistance in second test, compared to a control group with no AI	No significant improvement for either group. Learner feedback positive about AI software for education
33	Rudie et al. [51] USA	Neural network algorithm to interpret MRI images. Both radiologists and trainees interpreted images with and without AI to assess performance	Trainee’s got correct diagnosis 55% with AI and 30% without. Improvement not seen for specialists, implying educational value
	**Section 3: Interactive learning**		
34	Aldeman et al. [52] Brazil	Machine learning and decision tree based interactive online platform to teach renal pathology	Universalises access to specialist education with interactive element
35	Li et al. [53] Hong Kong	Machine learning and natural language processing to create chatbot for anatomy education. Trialled in small group of medical students	Improved student confidence with anatomy from 2.1 to 3.84 on 5-point Likert scale. Students valued the chatbot and felt confident interacting with it
36	Maicher et al. [54] USA	Natural language processing alongside neural network to generate virtual patient system for medical students to practice history taking	AI assessment correlated highly with expert-led assessment, at 89.3% accuracy. Gives students automatic feedback following practice
37	Maicher et al. [55] USA	Update natural language processing virtual patient system allowing verbal communication	AI assessment improved from previous model to > 90% correlation compared to expert-led
38	Ruberto et al. [56] Canada	AI dynamically adapts clinical simulation difficulty based on physiologic parameters to optimise cognitive load	Demonstrated feasibility. Positive feedback from participants about learning value
39	Wang et al. [57] China	Natural language processing online platform for students to work through clinical cases	Using platform improved assessment performance from 69.9% to 85.6% for students
	**Section 4: Text interpretation**		
40	Cianciolo et al. [58] USA	Machine learning algorithm assessment of diagnostic reasoning essays using a language model	AI assessment correlated closely with expert, with less case-specificity and variability
41	Neves et al. [59] USA	Machine learning interpretation of feedback to trainees	AI assessment had accuracy of 74.4–82.2% compared to expert assessment
42	Ötleş et al. [60] USA	Natural language processing interpretation of feedback to trainees	AI assessment had accuracy of 83%

### Classifying Emergent Applications of AI in Medical Education

The included papers have been grouped into four emergent categories for analysis. These categories are surgical skills, radiology, interactive learning, and text interpretation. The category of surgical skills represents the majority of included articles (*n* = 30, 71%) and is primarily focussed on articles addressing AI augmentation of surgical simulation. The radiology category (*n* = 3, 7%) included articles utilising AI algorithms to improve radiologic image interpretation. Interactive learning (*n* = 6, 14%) included novel innovations using AI to replicate interaction with an educator, such as reactive online platforms. Finally, the category of text interpretation (*n* = 3, 7%) considers research applying AI to analyse written work for educational purposes.

#### Category 1: Surgical Skills

The main theme identified within this category (Table 3) is the ability of artificial intelligence to improve surgical training through high yield simulations that build surgical skills with minimal patient risk. Increasing public scrutiny and demand for accountability has contributed to the transition to competency-based surgical training with a focus on safety rather than the previous time-based model [24]. Traditionally, surgical training under the apprenticeship model was vulnerable to subjective assessment and ineffective or delayed feedback [24]. Therefore, the gaps to address in this process are rapid and accurate feedback, and objective assessment whilst minimising clinical resource consumption. Integrating AI into surgical simulation is a novel educational approach which has the potential to meet all these demands. However, without supervision and feedback, simulations offer easily measurable parameters such as time to completion which do not reflect competence or facilitate technical improvement [43]. The included studies support the role of AI in enhancing competency-based training alongside traditional expert-led teaching.

AI algorithms can improve the educational value of benchtop trainers by evaluating granular elements of surgical skill and providing feedback by contrasting with established standards. Analysis of kinematic metrics is one method to achieve this. AI algorithms reliably differentiate between expert and novice surgeons using data from wearable hand sensors and surgical tools [24, 44, 46]. Contrasting measured metrics with expert standards highlights areas for improvement. Studies from Kowalewski et al. [32] and Oquendo et al. [39] indicate strong correlation between AI and expert assessment using data from wearable sensors and surgical tool sensors respectively. AI interpretation of kinematic data therefore offers immediate and accurate feedback and assessment with fewer resources than expert observation. However, motion tracking sensors are relatively expensive, therefore potentially limiting implementation in the operating theatre due to sterility requirements [32]. Furthermore, whilst kinematic metrics measure simple tasks such as suturing, they do not necessarily offer detailed insight into holistic surgical performance. An alternative approach that is both more holistic and accessible involves video recording analysis. AI algorithms can accurately evaluate surgical skills from video footage, providing comparable insights to kinematic metric analysis [21, 23, 28]. Various algorithms applied to the same footage from robotic surgical simulations accurately discerned expert from novice [20, 25, 29, 31, 38, 45]. Beyond simulation, Lavanchy et al. [33] achieved the same result with non-simulated intraoperative footage. Hence, AI analysis of simulation or intraoperative footage can precisely evaluate skill and provide individualised feedback. Since video footage is routinely available for laparoscopic and robotic surgeries, the technological step is less than that required for kinematic sensors.

Furthermore, AI readily and accurately assesses performance on VR simulators [19, 42, 47]. AI algorithms identify specific metrics that contribute to performance and translate these into individualised feedback for learners [22, 36, 40]. For example, Bissonnette et al. [22] found that experts applied less force to surrounding tissue and more often utilised bimanual or two-handed skills. These conclusions are presented to learners as specific areas for improvement. One well developed example of AI augmented VR simulation is the ‘Virtual Operative Assistant’ (VOA). First described by Mirchi et al. [35], the VOA is based on a machine learning algorithm which assesses performance and provides goal-oriented feedback on a neurosurgical VR simulator. Learners receive immediate feedback including a visual representation of their performance across various metrics compared to benchmarks. The VOA therefore leverages the widely demonstrated ability for AI to distinguish expert from novice and translates this into directive feedback. Ledwos et al. [34] generated learning curves for trainees using the VOA to track their progress across metrics and offer visibility over development. For example, instrument tip distance remained stable for expert surgeons on serial simulations but reduced significantly amongst novices, implying that this metric is tied to experience. Similarly, the expert group demonstrated dynamic adaptation through increased force applied intraoperatively over serial trials, which was not seen amongst novices. These examples represent specific goals for future learning. In a randomised controlled trial, the VOA resulted in significantly improved performance scores on surgical simulation compared to VR simulation without AI feedback [26]. Similarly, Su Yin et al. [43] describe a dental VR simulator that provides video feedback highlighting specific instances where performance deviated from benchmarks, alongside suggestions for improvement regarding force application or distance between tools. In a trial, the group utilising this simulator with AI assistance demonstrated higher post-training performance and greater improvement from baseline that the group using VR simulation without AI assistance [43].

Intelligent tutoring systems (ITS) extend the role of AI in surgical training by providing personalised instruction tailored to the learner’s experience level and weaknesses [30]. This is beneficial to beginners learning surgical techniques and those more proficient looking to hone skills. Such systems have been implemented and demonstrate comparable efficacy to expert-led teaching [37]. Nakawala et al. [37] developed an ITS to teach thoracocentesis using a machine learning algorithm which recognises tools and guides learners through the procedure. Another ITS developed for orthopaedic surgical skills provides haptic guidance for drilling into bone based on an algorithm trained to recognise expert surgical skill [27]. Yang and Shulruf [48] demonstrated improvement in medical student suturing skill using AI over expert-lead teaching. Students practiced suturing and received individualised AI feedback on metrics including force application, suture tension and distance. This data also informed targeted discussions with clinical tutors. This AI approach facilitates self-directed learning with clear instruction for improvement whilst ensuring supervisor resource is utilised efficiently. Expert supervision cannot gauge parameters such as the force and tension applied. Most feedback is therefore based on the end result and not the process, and therefore not targeted at points of deviation. The AI group reported higher learner confidence with the suturing task in addition to improved objective performance [48].

#### Category 2: Radiology

Widely perceived as the medical field most vulnerable to the displacing effects of AI, radiology is simultaneously well placed to embrace the education benefits of AI innovation. These developments have been employed to facilitate improved education for trainees. For example, Rudie et al. [51] showed that an AI deep learning algorithm significantly improved trainee likelihood of reaching the correct diagnosis when interpreting brain MRI (55% vs. 30% without AI, p < 0.001), a benefit that was not observed for trained specialists, highlighting the educational potential of AI. Two studies explored the educational role of AI in X-ray interpretation among medical students. Cheng et al. [49] demonstrated significant post-training improvements in hip fracture identification in the AI training group, particularly for students with lower baseline scores. Conversely, Dao et al. [50] did not find a significant performance difference between the AI group and control for chest X-ray interpretation but did report positive feedback from students on ease of use and perceived educational value of the AI assistant. The divergent results are partially explained by the reduced diagnostic accuracy of the chest X-ray algorithm as it attempts to identify multiple findings rather than the binary task of fracture identification. Furthermore, the medical students identifying hip fractures trained with 100 cases compared with only 15 in the chest film trial. Nonetheless, both trials had small sample sizes of approximately 30 students which limits generalisability of findings. Despite these limitations, these trials indicate AI’s potential for enhancing radiology education. AI training helped students recognise radiological features of hip fracture and resulted in sustained improvement in performance [49]. By replicating the guidance traditionally offered by expert supervision, AI could increase access to training, promote self-directed learning and reserve supervisor resources for more complex and targeted educational tasks.

#### Category 3: Interactive Learning

Artificial intelligence can universalise access to high quality interactive learning. The ability to dynamically engage with an educator in medical training encourages exploration of learner understanding with immediate correction and explanation. However, the disruption introduced by the COVID-19 pandemic highlighted the need to adapt medical training for distance learning. AI has facilitated the development of interactive online platforms for education, offering instantaneous explanation and feedback [52]. A consistent strength of these platforms is the comfort learners feel using AI. Learners report increased confidence practicing clinical skills or asking questions, seemingly due to less fear of making mistakes and affecting their standing with assessors [53]. This is demonstrated with an interactive AI chatbot for anatomy education, which uses natural language processing to translate extensive anatomy knowledge into conversational outputs, answering learner questions and offering explanations [53]. Medical students report increased confidence with anatomy following training using this chatbot [53].

Other similar AI applications include virtual standardised patients, which use natural language processing to facilitate realistic interactions for students to practice history taking [54]. The latest iteration of virtual patients are verbally interactive, while also allowing simultaneous assessment of the student’s performance and the provision of feedback following the interaction [55]. Similarly, Wang et al. [57] developed an interactive platform for medical students to practice clinical reasoning. Students showed significant improvement in reasoning following a brief learning session using this AI platform which requires learners to work through the history, examination findings and investigations of realistic cases [57].

A further strength of interactive AI platforms is increasing access to high-quality education which otherwise is not universally available due to resource distribution. Aldeman et al. [52] describe an interactive AI platform to teach the highly specialised subject of renal pathology. This system is trained on a machine learning algorithm to recognise characteristic features of glomerular disease. Students work through cases and receive immediate feedback with explanations [52]. Such platforms extend access to interactive education on specific topics.

#### Category 4: Text Interpretation

A further novel application of AI in medical education is the ability of AI to analyse large amounts of text. This makes tasks that are time and resource heavy, such as assessing essays, more accessible for educators. For instance, machine learning algorithms can evaluate diagnostic reasoning essays as accurately as human experts but are faster, more consistent and less prone to case specificity [58]. An additional application of using AI to analyse text is ensuring high quality feedback. Effective feedback is essential for learners to improve [59]. High quality feedback needs to be specific and actionable, incorporating opportunities for learner self-refection and should provide a scaffold for future improvements [59]. Feedback to medical trainees is relatively infrequently given and often poor quality [59]. Low quality feedback is demotivating and negatively impacts learning. Manually ensuring feedback to trainees is of sufficient quality would be a challenging and time-consuming process. AI has demonstrated the ability to reliably recognise the hallmarks of effective feedback. Neves et al. [59] trained and validated a machine learning algorithm to recognise high quality feedback, whilst Ötleş et al. [60] achieved the same goal using a natural language processing model. Both approaches rapidly screen and characterise feedback with accuracy similar to human experts. Whilst these are small studies, they highlight the future application of AI in improving education through feedback. The primary application will be identifying supervisors that routinely provide low-quality feedback and offering targeted education to improve this feedback. Alternatively, these models could automatically screen feedback prior to submission to encourage the supervisor to craft sufficiently high quality feedback.

## Discussion

From a systematic search and review of 1325 records, 42 were included in this scoping review of artificial intelligence in medical education. There has been a rapid growth in publications on this subject in recent years, a trend which is likely to continue [16]. This signals increased interest in the potential applications of AI in medical education. A range of AI methods and methodological approaches were applied in the included studies suggesting current uncertainty about the optimal approach to researching this novel topic. The discussion will focus on prominent themes derived from our review.

### Surgery

The majority of included studies centred on AI applications in surgical education. The initial functions distinguished the surgical skills of experts from novices in simulation, with AI demonstrating comparable accuracy to expert-led assessment for this task. In both cases, AI exhibits the ability to recognise elements of surgical competence. This has since translated into comprehensive systems for teaching and refining surgical practice which provide detailed feedback to scaffold improvements. There are a number of potentially valuable future applications based on the included reports, comprising ensuring basic levels of competency, highlighting trainees for additional targeted support, and facilitating self-directed learning.

As surgery evolves, trainees must gain competence in a greater number of increasingly complex procedures including robotics and minimally invasive surgery [61]. These expectations continue to grow despite barriers to acquiring expertise including work-hour restrictions, service pressures reducing clinical supervision and a complex medicolegal environment that is less receptive to trainee participation in care [61, 62]. This challenge is reflected in studies showing that surgical trainees are not invariably performing core procedures independently at the conclusion of training [63]. The COVID-19 pandemic further steepened this learning curve by reducing opportunities to treat patients [64]. This highlights the importance of increasing the efficiency of surgical education. AI-augmented simulation offers a reliable method to develop skills whilst minimising risk to patients [65]. Traditional surgical training involves an apprenticeship model, where surgeons learn through task repetition and graded independence under expert supervision. Feedback is centred around semi-objective assessment such as the Objective Structured Assessment of Technical Skill (OSATS) [66]. In this apprenticeship model, exposure to a certain number of procedures deems trainees ready to attempt a task in the operating theatre. There is no minimum demonstrated competency required to begin operating on patients, and correspondingly no guarantee that trainees possess the foundational skills to operate safely. The ability of AI to accurately assess surgical skill via simulation can be used to develop and implement competency minimums, a likely feature of the transition to competency-based training. AI therefore has a role to bridge the gap between novice and suitable minimum competence to operate [35]. Similarly, AI-augmented simulation can identify trainees requiring additional supervision early in training. In the current system, subjective assessment and discontinuous supervision may leave inadequate performance unaddressed. Consistent assessment using AI will remove barriers to identifying the struggling trainee and instituting early support.

AI will complement traditional methods of surgical training by offering unique perspectives into discrete elements that contribute to surgical skill [36]. Novel suggestions can be generated by AI algorithms that recognise the characteristics of expert skill and identify the specific factors that either contributed or detracted from performance compared to this standard [35]. These factors are less likely to be recognised by clinical assessment which focusses on overall performance or final outcome, parameters that can conceal deficiencies and are insufficiently specific to guide improvement [65]. Examples of specific performance metrics that AI can follow range from needle angle during suturing, to complex bimanual tool use during tumour resection [24, 34]. This highlights the multifaceted nature of expert surgical performance, and that each component requires direct attention.

Critical self-assessment is an important skill for surgeons, who must commit to acknowledging weaknesses and specifically addressing them through self-directed learning [44]. AI will make self-directed learning more efficient by removing barriers to accurate assessment and immediate feedback. Trainees can incorporate feedback in an iterative approach and track longitudinal progress reliably using consistent standards.

### Learner Comfort

The reported comfort of learners when interacting with AI in medical education is a significant observation. Digital systems have previously been noted to invoke negative emotional responses in learners, something assumed to be due to the lack of appropriate affectation and inability to recognise emotional needs [67]. However, this was not apparent in any included studies in this review. Instead, students consistently reported increased confidence when asking questions of AI, due to the perceived lack of the risk of embarrassment. Whether this reflects a cohort of learners that are generationally more confident with AI, or improvement in AI affectation is uncertain. An important conclusion is that AI implementations in education should not be universally monitored by human educators as this may undermine learner freedom to express uncertainty.

### Humanistic Qualities

If AI is to be widely implemented in education, it is important to recognise that AI has not demonstrated the ability to assess or embody fundamental humanistic qualities. Essential medical skills such as complex communication, teamwork and leadership were not addressed in any included studies. Effective medical practice also requires navigating nuance and uncertainty, which are not natural qualities of AI. This underscores the importance of retaining human-led medical education alongside AI. Despite widespread concern about being replaced by AI, successful use of AI in the future of medical education is more likely to resemble an adaptation to work alongside AI [68]. AI can streamline processes for educators facing increased workload pressure and more complex content. This will likely manifest in the reprioritisation of skills required by human educators. Qualities such as motivating learners and providing mentorship remain the domain of teachers [2]. However, deferring ‘mundane’ tasks to AI such as addressing learner queries may have unintended consequences, such as missing the deeper distress lying behind a simple question posed by a learner requiring further support [2].

### Risks and Weaknesses of AI

AI may be thought to be infallible, with the ability to perform tasks more rapidly and accurately than humans. However, as AI is implemented widely, it is becoming increasingly apparent that it carries its own set of vulnerabilities [69]. One significant issue is the ‘black box’ element of AI systems; when AI does fail, the reasons for errors often remain obscured due to the lack of transparency in how AI makes decisions. A relevant consideration in the health education context is the risk of entrenching the existing bias that is prominent within healthcare datasets used to train AI [16]. AI was intended to be less prone to bias than human decision makers [69]. Unfortunately, any bias present in programming data will persist and can amplify existing discrimination [2]. For example, AI algorithms for detecting skin cancer are primarily trained on data from light-skinned individuals and may be less likely to recognise melanoma on darker skin [70]. In the education context, this bias may be unwittingly passed onto learners [71]. Educators implementing AI must be critically aware of these risks and take proactive steps to avoid exacerbating disparities. This demands further work into ensuring AI systems used in medical education are transparent, auditable and free of harmful biases.

Current strategies to mitigate these risks include actively diversifying training datasets, employing transparent algorithms, and incorporating auditability or explainability features in AI tools. Future research and practice should prioritise the systematic evaluation of AI algorithms for fairness and accuracy across diverse learner populations. Furthermore, involving multidisciplinary teams—including educators, ethicists, and computer scientists—in AI development and implementation can ensure these risks are appropriately addressed, enhancing both the fairness and reliability of AI in medical education.

While AI performs well with a simple and familiar task such as identifying a fracture or differentiating expert from novice, it can struggle with more complex assessments. AI algorithms demonstrate ‘brittleness’ in their inability to adapt to new variables [69]. This is a barrier to generalisability of the applications discussed in this research. For instance, while distinguishing between an expert and a novice in surgical training is useful, a more valuable system would deliver a nuanced understanding of the varied layers of intermediate performance. The potentially narrow scope of AI’s ability might become more apparent in larger studies with more robust or comprehensive outcome measures.

### Strengths and Limitations of This Study

The strength of this research lies in the thorough systematic search and supplemented by manual searching of relevant reference lists. This ensured that a good number of relevant records were extracted for analysis. Limitations include the heterogenous nature of current reports on AI in medical education, which made synthesis of the evidence difficult and precluded quantitative meta-analysis of outcome measures. Additionally, English language databases were exclusively used, neglecting research published in other languages such as Chinese and Japanese. Despite a thorough search, there is a possibility that relevant papers may have been missed.

Given the emerging nature of research in this field, most included records were proof of concept or validation studies. A minority of studies (9, 21%) had achieved routine implementation of AI as an educational intervention and assessed educational outcomes. These trials were non-randomised, with small samples and conducted at single centres, all of which limit generalisability.

There is a strong focus on postgraduate learning or clinical training using artificial intelligence in this review. Given the systematic nature of the search, this is likely to be reflective of the current range of evidence of AI applications in advancing learning and teaching in areas such as surgery and radiology. Further research into undergraduate applications of AI will be valuable.

One limitation of this study is the search period concluding at the end of 2022. Several recent reviews have explored aspects of artificial intelligence in medical education [72–75]. However, none have explicitly highlighted influences on surgical education, significantly expanded upon or altered the conclusions of our current review. These recent publications primarily provided broad overviews of the existing AI landscape, charted attitudes towards AI among learners, or discussed conceptual rather than practical implementations of AI. For example, Tozsin et al. [72] conducted a systematic review with a search period until March of 2023 and Gordon et al. [73] conducted a scoping review including articles until August 2023. There is no indication of any new information in the intervening time that would meaningfully change the conclusions drawn in this article, or alter our conclusions regarding the impact on surgical or radiology education. Furthermore, these reviews include many studies assessing learner attitudes or knowledge of AI. Thus, our review maintains its unique contribution by explicitly evaluating current AI applications, emphasising concrete educational outcomes, and outlining specific, actionable recommendations for integration into medical education.

### Future Directions

This review has identified a number of avenues for future research on the use of AI in medical education, such as surgical skill training. Firstly, larger controlled trials are essential to validate AI interventions against standard educational methods and clearly delineate their impact on learners. Future studies should explicitly measure validated educational outcomes such as skill retention, accuracy in procedural tasks (e.g. using OSATS scores), or longitudinal clinical performance. It is crucial to evaluate whether AI-driven improvements translate into clinical competence and patient care quality beyond controlled simulation environments. Acceptability and cost-effectiveness of AI interventions must also be explored to determine feasibility for widespread integration into medical curricula. With regard to acceptability, it will be important to focus on whether learners continue to feel positively about interacting with AI. Finally, since various AI methodologies (e.g. neural networks, machine learning, natural language processing) have shown comparable efficacy, future studies should aim to identify the optimal methods for specific educational outcomes or tasks, thus informing tailored implementation strategies.

## Conclusion

This review demonstrates that AI holds significant potential to reshape medical education, by enhancing procedural skills, personalising learning experiences, and broadening access to educational resources. However, most existing evidence is preliminary, derived from small scale or proof of concept studies, limiting generalisability. To advance the field, future studies should prioritise rigorous evaluation of AI interventions using validated educational metrics, including learner confidence, clinical competence, and patient-centred outcomes. Practical steps for educators include integrating AI tools to provide precise, individualised feedback and employing AI-driven simulations to bridge competency gaps. This will free educators to focus on mentorship, communication, and other humanistic roles. Researchers and educators should explicitly address AI limitations such as algorithmic bias and transparency by ensuring diversity in training datasets and promoting auditability. As AI continues to mature, thoughtful and evidence-based integration of AI promises to reshape medical education, particularly in advancing learning in surgery and radiology, by fundamentally redefining the educator role and enhancing learner experiences.
